# Case of Contused Urethra, &c.

**Published:** 1809-05

**Authors:** 


### *To the Editors of the Medical and Physical Journal*

Gentlemen,

In looking over your last Number, I was forcibly struck with a case, related very minutely by Dr. Bellamy, of an accident which had occurred to a seaman on board His Majesty's Ship Glory, and which ended fatally.

My attention was peculiarly called to this case, from the recollection of a circumstance, in many respects similar, which happened in this town some years ago. I shall simply relate the case, to lessen those apprehensions which the issue of Dr. Bellamy's may induce.

About the middle of May, 1801, as a nobleman of considerable importance in the state was riding in Hyde Park, his horse taking fright, ran away with him;—from the suddenness and rapidity of the animal's motion, the rider lost his stirrups and seat at the same time, and was forcibly thrown forward on the pommel of the saddle, where he remained some time, the horse plunging and galloping violently. The gentleman being a good horseman, several times attempted to regain his seat, but was as often thrown forward; when finding himself much injured he threw himself on the ground, and having obtained speedy assistance, was carried to his own house at no great distance.

A surgeon of eminence (now no more) saw him in the first instance, and found that very considerable contusion had happened along the course of the urethra, and that the penis and scrotum were much tumefied; the latter indeed, appeared to be filling fast with extravasated blood and urine, which had insinuated themselves into the cellular membrane, in consequence of the passage to the bladder by the urethra having been almost entirely obliterated by the accident. Under these alarming appearances, three other surgeons arrived, who were equally embarrassed as the first, with regard to the mode of procedure in this very distressing situation of the patient.

As a precautionary measure, the patient was blooded, took an aperient, and a saturnine lotion was applied to the injured parts; several attempts were made to explore the passage of the urethra but in vain; in the evening, the appearance of the parts was still more formidable; the scrotum at this time was enlarged to the size of a child's head, perfectly black; and it was evident that all the urine secreted passed into it. Under these alarming circumstances, very late at night, (ten hours after the accident) Mr. Cline was called in, and on consultation it was determined that an opening into the bladder presented the only chance of saving the life of the patient for the present, whatever might be the result of the mischief already occasioned.

Mr. Cline, with his accustomed dexterity, immediately performed this operation, by making a lateral incision in the perinæum, (as in cases of lithotomy, but without the usual guide by staff or sound); and having punctured the bladder, urine issued from the wound. The patient, who displayed uncommon firmness on the occasion, was put to bed, and an opiate was administered. In the morning the appearances, though still very alarming, were evidently more favourable; the urine continued to flow through the artificial opening, and the scrotum had not increased in size. All immediate danger had subsided, and it was hoped that when absorption of the fluid contained in the scrotum took place, that an opportunity might present itself of discovering the passage into the bladder by the urethra; all attempts at which, under the present circumstances, proved completely abortive. These expectations were verified in the event.

The patient was enjoined a strict regimen, due attention was paid to his bowels, and an opiate generally administered at night. In the course of a fortnight, the absorbing process presented a curious phenomenon. The whole surface of the abdomen, and from the pubis to the chin, displayed extravasated fluid in all its shades upwards. No untoward symptom had occurred; the urine continued to pass through the puncture. A portion of the urethra was discovered, and every day more of that passage was found out; till at length, before a month had expired from the time of the accident, a few drops of water discharged itself through the urethra, and afterwards continued to pass through that channel; the external opening was allowed to heal, the parts were reduced to their natural size, and suffice it to say, that in the middle of July, not quite two months from the time of the accident, the nobleman in question proceeded to a distant part of the Empire, (where he filled a high judicial situation) without the slightest inconvenience; nor did he ever after experience any, although he survived the operation three years, and then died of a bilious disorder.

The case detailed by Dr. Bellamy, resembles the above in some particulars; if the relation of it should prove useful to practitioners in surgery, my purpose will be completely accomplished. I am, &c.

*London, March,* 1809. VERAX.*

* The name of the author, which is highly respectable, is in the possession of the Editors.

